# Developmental Fluoxetine Exposure Normalizes the Long-Term Effects of Maternal Stress on Post-Operative Pain in Sprague-Dawley Rat Offspring

**DOI:** 10.1371/journal.pone.0057608

**Published:** 2013-02-21

**Authors:** Liesbeth Knaepen, Ine Rayen, Thierry D. Charlier, Marianne Fillet, Virginie Houbart, Maarten van Kleef, Harry W. Steinbusch, Jacob Patijn, Dick Tibboel, Elbert A. Joosten, Jodi L. Pawluski

**Affiliations:** 1 University Pain Center Maastricht, Department of Anesthesiology/Pain Management, Maastricht University Medical Center, Maastricht, The Netherlands; 2 School for Mental Health and Neuroscience, Maastricht University, Maastricht, The Netherlands; 3 University of Liege, GIGA-Neurosciences, Liège, Belgium; 4 Laboratory of Analytical Pharmaceutical Chemistry, Department of Pharmacy, CIRM, University of Liège, Liège, Belgium; 5 Intensive Care and Department of Pediatric Surgery, Erasmus MC-Sophia, Rotterdam, The Netherlands; Radboud University, The Netherlands

## Abstract

Early life events can significantly alter the development of the nociceptive circuit. In fact, clinical work has shown that maternal adversity, in the form of depression, and concomitant selective serotonin reuptake inhibitor (SSRI) treatment influence nociception in infants. The combined effects of maternal adversity and SSRI exposure on offspring nociception may be due to their effects on the developing hypothalamic-pituitary-adrenal (HPA) system. Therefore, the present study investigated long-term effects of maternal adversity and/or SSRI medication use on nociception of adult Sprague-Dawley rat offspring, taking into account involvement of the HPA system. Dams were subject to stress during gestation and were treated with fluoxetine (2×/5 mg/kg/day) prior to parturition and throughout lactation. Four groups of adult male offspring were used: 1. Control+Vehicle, 2. Control+Fluoxetine, 3. Prenatal Stress+Vehicle, 4. Prenatal Stress+Fluoxetine. Results show that post-operative pain, measured as hypersensitivity to mechanical stimuli after hind paw incision, was decreased in adult offspring subject to prenatal stress alone and increased in offspring developmentally exposed to fluoxetine alone. Moreover, post-operative pain was normalized in prenatally stressed offspring exposed to fluoxetine. This was paralleled by a decrease in corticosteroid binding globulin (CBG) levels in prenatally stressed offspring and a normalization of serum CBG levels in prenatally stressed offspring developmentally exposed to fluoxetine. Thus, developmental fluoxetine exposure normalizes the long-term effects of maternal adversity on post-operative pain in offspring and these effects may be due, in part, to the involvement of the HPA system.

## Introduction

Early life events can significantly influence the development of the nociceptive network and pain responses in later life [1]. Recently, maternal adversity in the form of perinatal stress and depression has been shown to affect not only the mother, but also the neonate. Moreover, perinatal maternal stress has been linked to increased nociceptive responses in the newborn [2]–[4]. For example, exposure to prenatal maternal stress increases a baby's response to a painful heel prick [2]. Perinatal maternal depression, often a stress-related disease, is a major condition which affects 20% of women [5]. However, little is known about the long-term impact of maternal adversity on nociception in offspring.

Preclinical work has shown that perinatal maternal stress results in increased pain sensitivity in adult rat offspring [6], [7]. Underlying mechanisms for such long-term effects of maternal adversity on nociception in later life are rather unknown. Clinical and preclinical studies have shown that intra-uterine exposure to high levels of glucocorticoids or maternal stress affect stress responses in offspring [2], [8]–[11]. Furthermore, a dysregulated hypothalamic-pituitary-adrenal (HPA) system has been related to alterations in pain responses [12], [13]; decreased cortisol levels have been linked to increased pain symptoms in patients with chronic pain [14]. Glucocorticoid receptors (GRs) are involved in pain processing given their presence on nociceptive neurons in the spinal cord [15], [16]. Moreover, these neurons show signs of activation when C-fibers are noxiously stimulated [17] and intrathecal application of a GR antagonist has been shown to attenuate neuropathic pain [18].

Selective serotonin reuptake inhibitor (SSRI) medications, e.g. fluoxetine, are the most common treatment for maternal depression and are prescribed to 5–10% of pregnant mothers [19]–[21]. SSRIs cross the placenta [22] and are transferred, via breast milk [23], to the infant. Developmental SSRI exposure increases serotonin (5-HT) levels in offspring and may affect the development of the 5-HT-pain inhibitory circuit [1]. Indeed, recent clinical research has shown that infants perinatally exposed to SSRIs have blunted pain responses to a heel prick at two days and two months after birth [24], [25], demonstrating an involvement of SSRIs on offspring nociception. Besides having a direct effect on the nociceptive system, 5-HT levels play a prominent role in the development of the HPA-axis [26], [27] and perinatal exposure to SSRIs can alter the developing HPA system [28], [29]. Therefore SSRIs may impact the developing nociceptive system indirectly via altering the HPA-axis.

The objective of this study was to investigate the effect of maternal stress on nociception in adult offspring. In turn, because SSRIs are commonly used for treatment of maternal stress-related disorders, we investigated the effect of developmental exposure to SSRIs on nociception in adult male offspring taking into account the involvement of the HPA system on these effects. To do this prenatally stressed offspring were exposed to fluoxetine during the perinatal period. Offspring were tested for basal nociception and post-operative nociception after hind paw incision during adulthood. Moreover, circulating corticosterone and corticosteroid binding globulin (CBG) levels were investigated. This work provides important information on the long-term impact of developmental exposure to maternal adversity and SSRI treatment on nociception and sheds light on the underlying physiology related to these changes.

## Materials and Methods

### Ethical Statement

All animal experiments were performed in accordance with the European Directive for the Protection of Vertebrate Animals Used for Experimental and Other Scientific Purposes (86/609/EEC) and adhered to the Guidelines for the Care and Use of Mammals in Neuroscience and Behavioral Research (National Research Council 2003). The protocol was approved by the Animal Ethics Board of Maastricht University in accordance with Dutch governmental regulations (approval IDs: DEC 2008-184 and DEC 2010-150). All surgery was performed under isoflurane anesthesia, and all efforts were made to minimize suffering.

### Animals

In this experiment a total of 20 Sprague-Dawley rats (Charles River Laboratories, France) were used. The animals were housed in a temperature (19–24°C) and humidity (55±15%) conditioned room with a 12∶12 light/dark schedule (lights on at 8:00 a.m.) with *ad libitum* access to tap water and rat chow. Breeding of the dams took place by putting one female and one male together in a wire mesh cage. Release of a vaginal plug was considered as gestation day (G) 0.

**Figure 1:**
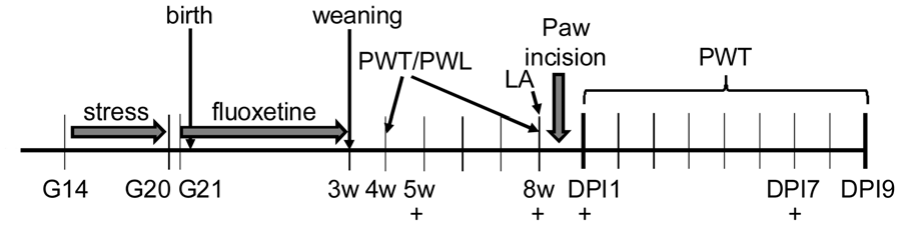
Schematic overview of the study. Pregnant dams were stressed three times per day for 45 minutes from gestational day (G) 14 until G20. The stressor consisted of restraint in a plastic cylinder and exposure to bright light. Fluoxetine was administered orally to the dams from G21 until the day of weaning at three weeks of age (3 w). After weaning, 2 male offspring from control/vehicle, control/fluoxetine, stress/vehicle and stress/fluoxetine dams were subjected to behavioral testing (*N* = 8/12 per group). Their basal paw withdrawal thresholds (PWT) to mechanical stimuli and paw withdrawal latencies (PWL) to heat stimuli were tested at 4 weeks, i.e. a juvenile age during development, and at 8 weeks, when animals were at a young adult age. Also, a locomotor activity (LA) test was performed at the age of 8 weeks. One day after the last behavioral basal test at 8 weeks, all offspring received an incision of the left hind paw, after which PWT were measured daily until 9 days post incision (DPI9). To measure serum levels of corticosterone and corticosterone binding globulin (CBG), tail vein blood was withdrawn at the age of 5 and 8 weeks and at DPI1 and DPI7 (+).

On G14, dams were randomly assigned to stress (*N* = 9) or control (*N* = 11) groups. Dams in the stress group were individually restrained three times a day (at 8:00 a.m., 11:30 a.m. and 15:30 p.m.) for 45 min in a plastic cylinder under bright light on G14-G20 [11], [30], [31]. Stress during this period of pregnancy can result in postpartum depressive-like behavior in the dam [32], [33]. The day after the last stress exposure (G20) dams were randomly assigned to one of the two treatment groups: fluoxetine (two doses of 5 mg/kg per day) or vehicle, resulting in a total of four groups of dams: 1. Control+Vehicle (CV; *N* = 5), 2. Control+Fluoxetine (CF; *N* = 6), 3. Prenatal Stress+Vehicle (SV; *N* = 4), 4. Prenatal Stress+Fluoxetine (SF; *N* = 5). After weaning at P21, two male offspring per litter were housed together in standard cages and used for behavioral testing, resulting in group sizes of *N* = 8–12. Offspring were weighed at the age of 8 weeks and at 1, 7 and 9 days after surgery. For a timeline of the experiment see Figure 1.

### Fluoxetine treatment

During G14–20 dams were trained for oral ingestion of the medication treatment. Dams were fed 1/9^th^ of a vanilla wafer-cookie (Croustifondante, Delacre, Belgium), filled with saline [34]. During feeding dams were not separated from the litter. At G21 treatment was started and dams were fed two times per day (at 9:00 a.m. and 14:00 p.m.) a cookie filled with fluoxetine (Flagron, Belgium) dissolved in vehicle (50% propylenediol in saline; 5 mg/kg) or vehicle solution. Since maternal restraint stress results in postpartum depressive-like behavior, as mentioned before, dams were treated with fluoxetine from G21 until P21. Oral administration via a cookie was preferred instead of oral gavage or injections, which might induce pain and stress in the dam/litter. Since fluoxetine and its active metabolite norfluoxetine are passed to offspring via lactation [35], fluoxetine and norfluoxetine levels were determined at several time-points during lactation (see section 2.3). Exposure of offspring to fluoxetine occurred during a stage of neural development in rodents analogous to that of the third trimester in humans [36], when nociceptive fibers are present but in an immature stage [1].

### Fluoxetine and norfluoxetine determination

To determine fluoxetine and norfluoxetine concentrations in rat serum, blood samples were collected by quick decapitation from culled pups on P1, from one female pup on P7 and P14, and from the dam, one female and one male pup at P21. Although, behavioral testing was performed only in male offspring, female offspring were used for drug determinations to provide an indication of the drug levels in the pups at this age. This took place in every litter, where surplus pups were available. Blood samples were centrifuged at 10 000×*g* for 10 minutes and serum was collected. Drug concentrations were determined from serum using liquid chromatography coupled with mass spectrometry (LC-Chip-MS/MS) was used as previously described [37], [38]. Briefly, the chromatographic separation was achieved on a 1200 series LC-chip system (Agilent Technologies, Germany) using a Ultra High Capacity chip including a 500 nL trapping column and a 150 mm×75 µm analytical column, both packed with a Zorbax 80SB 5 µm C18 phase (Agilent Technologies). The mobile phase was composed of H2O/FA (100∶0.1, v/v) (A) and ACN/H2O/FA (90∶10∶0.1, v/v/v) (B) and used in gradient elution mode. Mass spectrometric detection was performed using a 6340 Ion Trap equipped with a nanoelectrospray ionization source operating in positive mode (Agilent Technologies, Waldbronn). Finally, an Oasis µElution MCX 96-well plate (Waters, UK) was used to prepare the samples for the analysis. 25 µL serum was needed per experiment and all conditions were performed in duplicate and back-calculated using a calibration curve. Mean drug levels show the presence of fluoxetine and norfluoxetine in offspring serum at the age of P1, P14 and P21 (Table 1) (N = 7–11/group). This shows that fluoxetine is transferred to offspring via lactation.

**Table 1:** Mean (±SEM) serum levels of fluoxetine (ng/mL) and norfluoxetine (ng/mL) during the postnatal period. (P = postnatal day).

	P1	P7	P14	Male P21	Female P21	Mom P21
Fluoxetine (ng/mL)	19.46 (±22.98)	2.44 (±2.90)	1.76 (±5.38)	2.07 (±3.98)	1.52 (±2.47)	30.65 (±59.14)
Norfluoxetine (ng/mL)	83.01 (±14.45)	16.32 (±17.33)	11.35 (±19.26)	27.41 (±21.03)	33.61 (±21.26)	184.63 (±104.66)

(*N* = 7–11).

### Nociception behavioral tests

Sensitivity of offspring to mechanical stimuli and thermal stimuli was tested at several time points: basal nociception was tested during development at 4 weeks of age and at the young-adult age of 8 weeks and post-operative hypersensitivity was tested daily in young-adult rats until 9 days after surgery; (Fig. 1). Sensitivity to mechanical stimuli was determined by measuring paw withdrawal thresholds (PWT) using plantar application of 10 von Frey monofilaments (North Coast Medical, Inc., California, USA) with logarithmically incremental stiffness (0.166, 0.407, 0.692, 1.202, 2.041, 3.63, 5.495, 8.511, 15.136 and 28.84 g). Animals were placed in Plexiglass cages on an elevated wire mesh floor and allowed to adapt to the cage for 30 min before testing. Starting with the lowest filament, each filament was applied five times with a duration of one second and inter-application interval of a few seconds to the plantar paw surface. Maximal applied force was defined by maximal withdrawal, i.e. when the animal showed five withdrawal responses. The mechanical force resulting in a 50% withdrawal frequency was considered the PWT and was calculated from a sigmoid curve (withdrawal frequency per filament vs. force log applied) by regression analysis (GraphPad Prism Version 4.00, CA, USA) [39].

Thermal sensitivity was determined using the Hargreaves thermal plantar algesia apparatus at the age of 5 and 8 weeks (Ugo Basile, Collegeville, PA, USA). After an adaptation period of about 30 minutes, an infrared laser beam was placed at the mid-plantar surface of the hind paw. The apparatus measures the paw withdrawal latency (PWL) from the infrared source (intensity of 70) to the nearest 0.1 s. Mean PWL from three measurements per paw (ten minute intervals) was measured with a cut-off time of 20 s. For post-operative testing of hypersensitivity, the Hargreaves apparatus was not used, since mechanical hypersensitivity is reported to be the main outcome for post-incision pain [40]. The researcher performing behavioral testing was blinded to grouping of the animals.

### Locomotor activity

Locomotor activity was investigated at 8 weeks of age in offspring to ensure there were no gross differences in locomotion between groups. Animals were placed individually in the center of a 50 cm×100 cm glass arena and their activity was recorded for 10 minutes. All animals were tested between 2:00 and 5:00 p.m. A video-tracking system (Anymaze, Stoelting) was used to measure the distance the animals travelled in the arena. The apparatus was cleaned with 70% ethanol and dried between rats.

### Post-operative pain at the age of 8 weeks: plantar hind paw incision

Plantar hind paw incision was performed at the age of 8 weeks, a young-adult age at which alterations in the nociceptive network organization and in hind paw incision pain have been evidenced following neonatal pain exposure [41]. After baseline nociceptive and locomotor activity testing, all male offspring received an injury to the left hind paw, i.e. plantar hind paw incision as a model for post-operative pain [40]. Animals were anesthetised with 3–5% isoflurane (Abbott Laboratories Ltd., Kent, U.K.), mixed with air enriched with 100% oxygen at a constant flow rate of 250 ml/min. Body temperature was maintained at 37.5°C using an automatic heating pad. After making a±1 cm longitudinal incision through the plantar skin and fascia, the plantaris muscle was elevated and incised at the midline as described previously [40]. Two mattress-sutures with 5–0 nylon on a PS-2 needle (Ethicon, San Lorenzo, P.R.) were used to close the wound. Hypersensitivity to mechanical stimuli after surgery was determined daily by measuring PWT to von Frey filaments until 9 days after surgery.

### Corticosterone analysis

To investigate effects of maternal stress and developmental fluoxetine on corticosterone levels in offspring in basal conditions and at one and seven days after hind paw incision, blood samples were collected from the tail vein. Blood samples were centrifuged at 10 000×*g* for 10 minutes and serum was collected. A commercially available corticosterone RIA kit (Corticosterone I^125^ for rats and mice, MP Biomedicals, NY, USA) was used to determine total corticosterone levels in duplicate serum samples. Interassay variability was 7.4%. The lowest detection limit of the kit was 7.7 ng/mL

### Corticosterone binding capacity

Corticosterone binding capacity was determined by measuring the level of CBG according to an earlier described method [42], [43]. Specifically bound CBG counts were calculated by subtracting the non-specific background counts from the average of total bound CBG counts. Interassay variability was 7.3%.

### Statistical analysis

All data shown were presented as means±standard error of the mean (SEM). Two-way analysis of variance tests (ANOVA) were used to analyze the effect of group (condition: stress/control, treatment: fluoxetine/vehicle) as independent variables on offspring weight, locomotor activity outcomes, PWT, corticosterone levels, and CBG levels as the dependent variable. Additionally, a mixed design ANOVA was performed with condition (stress/control) and treatment (fluoxetine/vehicle) as the independent variables and time as the repeated measure for CBG and corticosterone serum levels and separately for each paw for PWTs comparisons. Post hoc comparisons were performed for further analysis of single effect comparisons and utilized the Fischer's least significant difference (LSD) test. Correlations were conducted between CBG levels and PWT (log transformed). *P*≤0.05 was considered to be statistically significant (Statistica Version 9).

## Results

### Offspring weight

Total litter weight and the number of male and female pups was not significantly affected by maternal stress and/or fluoxetine exposure (0.17≤*P*≤0.97) (Table 2). There was a significant main effect of time (F(3, 108) = 592.36, *P* = 0.001) for all groups (Table 3) indicating that all animals gained weight during the experiment. However, no effect of stress or treatment was observed (0.38≤*P*≤0.87).

**Table 2:** Mean (±SEM) weights (g) of litters and number of male and female pups on postnatal day (PD 1).

	CV	CF	SV	SF
Litter weight at PD1 (g)	96.92 (±5.35)	97.25 (±4.54)	101.60 (±10.55)	92.00 (±7.00)
No. of males	6.80 (±0.49)	5.83 (±0.87)	5.50 (±0.65)	6.40 (±0.87)
No. of females	6.60 (±0.68)	7.83 (±0.75)	8.25 (±1.70)	6.60 (±0.93)

CV: control vehicle, CF: control fluoxetine, SV: stress vehicle, SF: stress fluoxetine, (*N* = 4–6/group).

**Table 3:** Mean (±SEM) weights (g) of male offspring.

Group	8 w weight (g)	DPI1 weight (g)	DPI7 weight (g)	DPI9 weight (g)
CV	323.98 (±6.54)	337.16 (±6.79)	366.71 (±6.85)	376.75 (±6.28)
CF	307.23 (±7.60)	322.39 (±5.77)	355.83 (±4.90)	365.08 (±5.02)
SV	321.11 (±7.52)	331.94 (±8.11)	366.71 (±9.36)	376.75 (±9.81)
SF	303.62 (±10.86)	315.98 (±11.69)	348.13 (±11.59)	365.00 (±11.67)

A statistically significant main effect of time was observed (F(3, 108) = 592.36, *P* = 0.001). DPI: days post incision, 8 w: 8 weeks, CV: control vehicle, CF: control fluoxetine, SV: stress vehicle, SF: stress fluoxetine (*N* = 8–12/group).

### Locomotor activity

To exclude locomotion problems, which might interfere with nociception behavioral testing, we evaluated overall locomotion and there was no effect of stress or treatment on the distance the animals travelled in the open arena (0.37≤*P* ≤0.74; Table 4).

**Table 4:** Mean (±SEM) distances that the animals travelled in the open arena at the age of eight weeks.

Group	Distance (m)
CV	12.69 (±0.66)
CF	13.74 (±0.87)
SV	12.68 (±1.26)
SF	12.20 (±0.58)

There were no differences in distances between the groups. CV: control vehicle, CF: control fluoxetine, SV: stress vehicle, SF: stress fluoxetine (*N* = 8–12/group).

### Basal nociception

**Figure 2:**
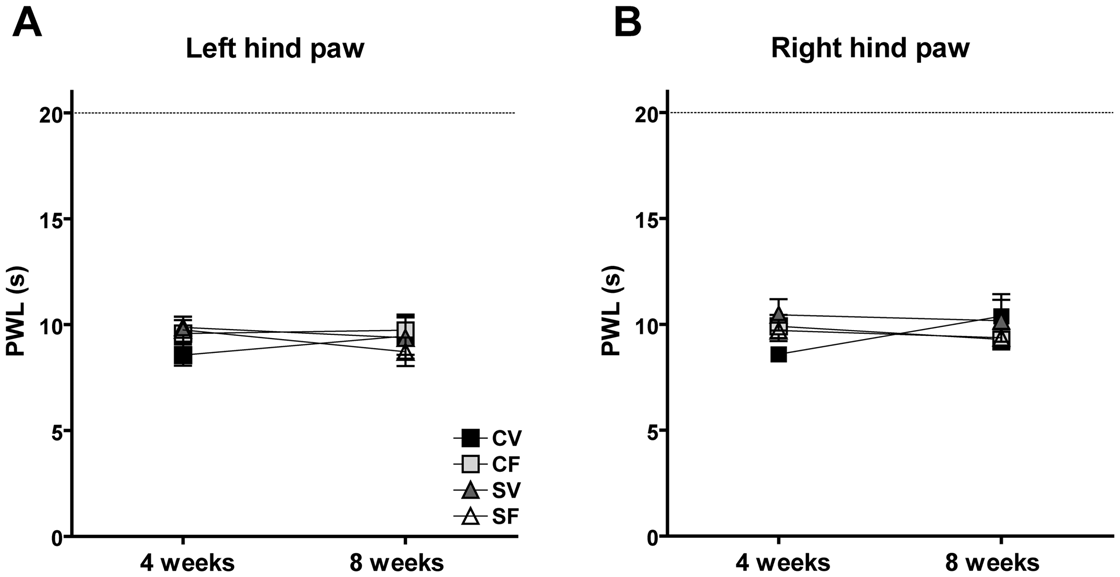
Mean(±SEM) basal nociceptive paw withdrawal latency (PWL) thresholds (s) to heat stimuli. There was no effect of maternal stress or fluoxetine on the PWL to heat stimuli at the left (**A**) or right hind paw (**B**) of male offspring at the age of 4 and 8 weeks. Also, there was no effect of time, since there was no difference in PWL between the age of 4 and 8 weeks. CV: control vehicle, CF: control fluoxetine, SV: stress vehicle, SF: stress fluoxetine (*N* = 8/12 per group).

**Figure 3:**
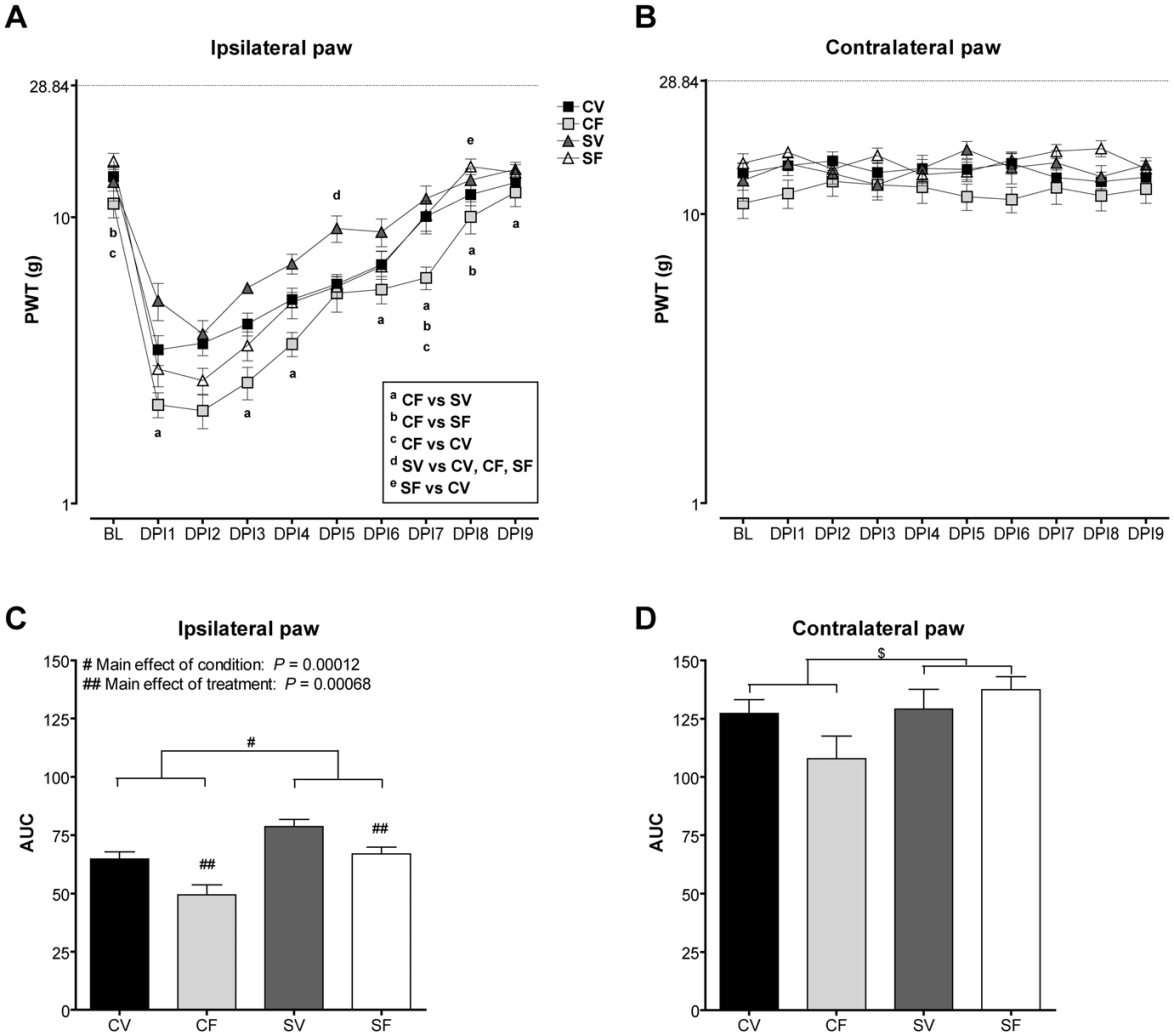
Mean (±SEM) PWTs in male offspring exposed to mechanical stimuli (g) after incision of the left hind paw. There was an overall interaction effect of time*condition*treatment observed for PWT to mechanical stimuli after incision. Also main effects of condition and treatment were observed. Significant differences were shown by post hoc correction and denoted by a, b, c, d and e symbols (A). Contralateral PWT were not affected by paw incision (B), since there were no differences observed between the groups. Area under the curve (AUC) analysis (C) of the ipsilateral PWT graphs in A, showed a main effect of condition (#) and a main effect of treatment (##). Contralateral AUC (D) showed a trend for a main effect of condition with control offspring having a tendency for lower PWT than stress offspring (^$^). DPI: days post incision, CV: control vehicle, CF: control fluoxetine, SV: stress vehicle, SF: stress fluoxetine (*N* = 8/12 per group).

There was no significant difference in PWL to thermal stimuli in offspring from mothers exposed to stress and/or fluoxetine at the age of 4 and 8 weeks (0.21≤*P*≤0.91; Fig. 2). There were also no statistically significant differences associated with condition or treatment in PWT to mechanical von Frey stimuli of male offspring at the age of 4 weeks (0.16≤*P*≤0.85; Table 5). The PWT at the age of 8 weeks served as the baseline value for post-operative hypersensitivity to mechanical stimuli and the analysis was therefore performed together with the post-operative hypersensitivity data (Fig. 3). At the age of 8 weeks, developmental fluoxetine exposure decreased PWT to von Frey stimuli (*P* = 0.0198; Fig. 3). However, since this decrease was only observed at the left (ipsilateral to the hind paw incision), but not at the right (contralateral) hind paw and was not observed at the age of 4 weeks, it is considered as not consistent.

**Table 5:** Mean (±SEM) PWTs to von Frey stimuli (g), reflecting sensitivity of the hind paws to mechanical stimuli at the age of 4 weeks.

Group	Left hind paw PWT (g)	Right hind paw PWT (g)
CV	12.14 (±1.68)	12.12 (±1.67)
CF	12.05 (±1.45)	12.25 (±1.45)
SV	13.92 (±2.29)	13.92 (±2.29)
SF	15.01 (±1.23)	14.68 (±1.20)

No differences were observed in PWT for the left and right hind paw between the groups. CV: control vehicle, CF: control fluoxetine, SV: stress vehicle, SF: stress fluoxetine (*N* = 8–12/group).

### Post-operative nociception

The effect of hind paw incision on PWT to mechanical stimuli was measured from one day post incision (DPI1) until DPI9. There was a significant interaction effect of time*condition*treatment (F(9, 324) = 2.41, *P* = 0.012) on PWT of the incised, i.e. ipsilateral, paw (Fig. 3A). CF offspring had the highest sensitivity to mechanical stimuli, i.e. the lowest PWT after surgery. Post hoc tests showed that CF offspring had lower PWT than SF offspring (^b^ *P* = 0.000095) and CV offspring (^c^ *P* = 0.019) at the baseline (BL), i.e. the day before surgery. Further post hoc tests showed that after incision CF offspring had lower PWT than SV offspring at DPI1, DPI3, DPI4, DPI6, DPI7, DPI8 and DPI9 (DPI1: ^a^ *P* = 0.019, DPI3: ^a^ *P* = 0.015, DPI4: ^a^ *P* = 0.0080, DPI6: ^a^ *P* = 0.0076, DPI7: ^a^ *P* = 0.000011, DPI8: ^a^ *P* = 0.0052, DPI9: ^a^ *P* = 0.042). Also, CF offspring had lower PWT than SF offspring at DPI7 (^b^ *P* = 0.00052) and DPI8 (^b^ *P* = 0.000020) and then CV offspring at DPI7 (^c^ *P* = 0.00073). Moreover SV had higher PWT than all other groups at DPI5 (^d^ 0.002≤*P*≤0.009). Also, there was a main effect of condition (F(1, 36) = 16.18, *P* = 0.00028) and treatment (F(1,36) = 10.19, *P* = 0.0029). There were no significant differences in contralateral PWT at any of the time points measured (0.13≤*P*≤0.79; Fig. 3B).

We also calculated the area under the curve (AUC) of the post-incision hypersensitivity (Fig. 3C-D). There was a significant main effect of condition on the AUC of the ipsilateral paw, with offspring exposed to maternal stress (SV and SF groups) showing higher AUC compared to offspring from control mothers (CV and CF groups) (F(1, 36) = 18.57, #*P* = 0.00012; Fig. 3C). Also, there was a significant main effect of treatment with developmental fluoxetine resulting in lower AUC compared to offspring exposed to vehicle treatment (F(1, 36) = 13.83, ##*P* = 0.00068; Fig. 3C). A trend toward a main effect of condition was observed on the contralateral paw with control animals having a tendency for lower AUC values (^$^ *P* = 0.056; Fig. 3D).

### Corticosterone levels

**Figure 4:**
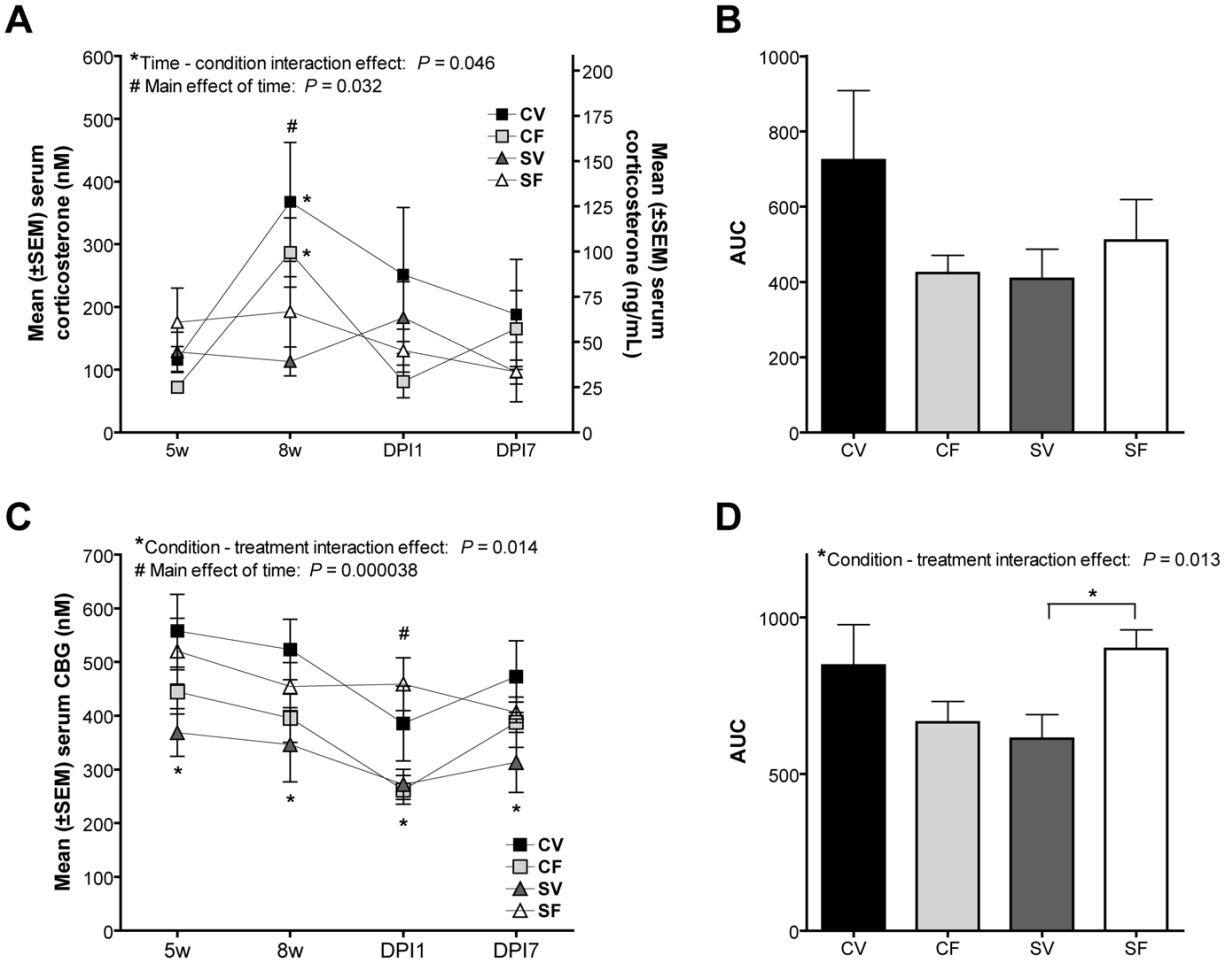
Mean (±SEM) and AUC of serum levels of corticosterone and CBG of male offspring. A time*condition interaction effect was observed in control offspring, but not stress offspring, having a higher corticosterone level at the 8 week time-point (**A**, *). There was a main effect of time on serum corticosterone levels and post hoc corrections showed that the levels at 8 weeks were significantly higher than levels at all other time-points (**A**, #). No effect of condition or treatment was observed on AUC of serum corticosterone levels (**B**). A condition*treatment interaction effect, with lower serum CBG levels in SV offspring, compared to SF and CV offspring (**C**, *). A main effect of time was observed and post hoc correction showed that this was attributable to the decrease in serum CBG at DPI1 (**C**, #). A significant interaction effect of condition*treatment was shown in AUC of CBG serum levels, with lower AUC values for SV offspring, compared to SF offspring (**D**, *). DPI: days post incision, CV: control vehicle, CF: control fluoxetine, SV: stress vehicle, SF: stress fluoxetine (*N* = 8/12 per group).

There was a significant time*condition interaction effect (F(3, 72) = 2.80, *P* = 0.046), with control offspring having higher corticosterone levels at the 8 week time point relative to stress offspring, regardless of fluoxetine exposure (*0.00018≤*P*≤0.019; Fig.4A). There was a significant main effect of time (F(3, 72) = 3.11, *P* = 0.032) on serum corticosterone levels. Post hoc correction showed that the main effect of time was confined to the increase in serum corticosterone levels at the age of 8 weeks (# 0.0057≤*P*≤0.028; Fig. 4A). Analysis of the AUC of the corticosterone curves, showed no significant differences (0.16≤*P*≤0.26; Fig. 4B).

### CBG levels

A statistically significant interaction effect of condition*treatment was noted (F(1, 24) = 7.08, *P* = 0.014; Fig. 4C). Post hoc tests showed that SV offspring had lower serum CBG levels than CV and SF offspring (* 0.031≤*P*≤0.045; Fig. 4C). There was a main effect of time (F(3, 72) = 9.01, *P* = 0.000038; Fig. 4C) which was mainly attributable to the decrease in serum CBG levels at DPI1 (# *P* = 0.0020; Fig. 4C). In the AUC, there was a significant interaction effect between condition and treatment (F(1, 31) = 6.89, *P* = 0.013) for CBG values, with higher CBG serum levels in SF offspring compared to SV offspring (* *P* = 0.037; Fig. 4D).

We also found significant positive correlations between CBG levels and basal nociception in the form of paw withdrawal thresholds (PWT) to mechanical stimuli of the ipsilateral (*r* = 32, *P* = 047) and contralateral paws on week 8 (*r* = 33, *P* = 037) and a tendency for a correlation in the contralateral paw on post incision day 1 (*r*  = 31, *P* = 051).

## Discussion

The present study demonstrates that prenatal maternal stress decreases, and developmental fluoxetine exposure increases, the intensity of post-operative hypersensitivity to mechanical stimuli in adult male offspring. Interestingly, post-operative hypersensitivity was normalized in offspring exposed to maternal stress in combination with fluoxetine exposure during development. Moreover, these effects were, in part, linked to changes in the HPA system.

### Prenatal stress and developmental fluoxetine exposure: effects on post-operative pain in adult offspring

Our findings show that maternal prenatal stress decreases post-operative hypersensitivity to mechanical stimuli in offspring at the age of 8 weeks. Although very little work has been done in this area, previous clinical work has shown that maternal stress can increase pain sensitivity in later life with increased somatic pain complaints at 18 months of age and recurrent abdominal pain in 13-year old children [3], [4]. Stress exposure in preterm born children affects their HPA-axis response to painful immunization [44] and their HPA system dysfunction has been related to mother behavior [45]. Previous preclinical work has also shown that maternal restraint stress increases inflammatory pain hypersensitivity in newborn and adult offspring [7], [46]. Thus, in contrast to our study, maternal stress has been shown to increase pain sensitivity. This difference may depend on the type of pain which is perceived, i.e. incisional pain in our study and inflammatory and abdominal pain in the above mentioned studies. A decrease in post-operative sensitivity to mechanical stimuli, as noted in our study, may be beneficial and indicate a faster recovery from paw incision. However, one study has shown that decreased post-operative pain might be detrimental as it can result in inadequate wound healing [47]. In the previously mentioned study, decreasing post-operative hypersensitivity to mechanical stimuli, after hind paw incision, with an analgesic treatment resulted in animals continually placing weight on the incised paw and consequently impaired wound apposition occurred [47]. We did not measure the degree of wound healing in the present study, but it is possible that there were differences in the rate of wound healing between offspring exposed to prenatal stress and control offspring.

We found that prenatally stressed offspring exposed to maternal fluoxetine treatment had normalized post-operative hypersensitivity to mechanical stimuli. Surprisingly, we observed that developmental fluoxetine exposure in the absence of prenatal stress increased post-operative hypersensitivity to mechanical stimuli in adult offspring. This highlights the need to investigate the effects of these medications using appropriate models of maternal adversity in order to draw suitable conclusions and clearly parallel the clinical situation. It has been shown that infants from depressed mothers treated with SSRIs have blunted pain responses at two days and two months of age [24], [25]. No other studies have investigated the effects of developmental SSRI exposure on post-operative hypersensitivity to pain or the long-term impact of perinatal SSRI exposure on pain sensitivity in any regard. However, developmental exposure to buspirone, a 5-HT1A receptor agonist, was shown to normalize the increased pain hypersensitivity in prenatally stressed adult offspring [7]. More research is needed to investigate how children, prenatally exposed to SSRIs, respond to painful stimuli in later life.

Although beyond the scope of this study, it should be noted that prenatal maternal stress and/or fluoxetine may have affected aspects of maternal care. Although previous work by our group has shown no marked changes in gross maternal care of offspring with prenatal maternal stress or fluoxetine treatment [11], we have also shown that fluoxetine exposure, but not prenatal stress, increases arched-back nursing of pups [38]. However, it is unknown how changes in arched-back nursing, without concomitant changes in licking behavior, may alter pain sensitivity in adult offspring.

### Involvement of the HPA system

In the present study we found that hind paw incision resulted in a decrease in serum CBG levels in adult offspring. Interestingly, offspring exposed to maternal prenatal stress alone showed overall lower serum CBG levels compared to prenatally stressed offspring developmentally exposed to fluoxetine and control offspring. This CBG profile is paralleled in their post-operative pain profiles, i.e. lower post-operative pain hypersensitivity in prenatally stressed offspring, and suggests a potential link between the HPA system and post-operative pain. Moreover, prenatally stressed offspring exposed to fluoxetine show serum CBG levels which are comparable with the levels of offspring from control mothers and thus, these levels may parallel effects of normalization of post-operative pain hypersensitivity by developmental fluoxetine exposure in prenatally stressed offspring. Fluoxetine exposure alone appeared to decrease serum CBG levels in adult offspring, as previously described [29], but did not parallel post-operative pain hypersensitivity. Therefore, there may be a relationship between circulating CBG levels and post-operative pain, at least in prenatally stressed male offspring. We did not find a significant correlation between overall CBG levels and pain sensitivity after paw incision. It is likely that a number of factors are mediating this relationship and it also may be that CBG levels at the point of the incision would better clarify the relationship between CBG and post-operative pain sensitivity.

SSRIs increase 5-HT levels in the newborn and 5-HT levels play a prominent role in the development of the HPA-axis [26], [27] and therefore SSRIs may impact on the nociceptive system indirectly via the HPA-axis. Interestingly this work suggests that serum CBG levels can be indicative of post-operative pain hypersensitivity, but only when fluoxetine exposure is not present. This is perhaps not surprising as clinical work has shown that the action of antidepressant medications may act to alter CBG levels [48]. Thus developmental exposure to SSRIs may differentially alter the function of CBG with regards to pain sensitivity.

We did not find an effect of hind-paw incision on circulating levels of total corticosterone levels. The absence of an effect of surgery on corticosterone levels in the present study is in line with previous work showing no increase in circulating serum corticosterone levels at 24 hours after open thorax surgery in a rat [49]. However, in humans, increased serum cortisol levels have been shown after major colonic and abdominal surgery [50], [51]. Discrepancies between these findings may be related to the severity of the surgical intervention that determines the increase in circulating corticosterone levels or to the timing of blood withdrawal after surgery.

### Prenatal stress and developmental fluoxetine exposure: effects on basal nociceptive thresholds in offspring

Besides a small effect of developmental fluoxetine on ipsilateral PWT at 8 weeks, we did not find any significant effect of prenatal stress and/or developmental fluoxetine exposure on basal nociceptive thresholds to mechanical and thermal stimuli in juvenile and adult male offspring. Although these parameters were previously not specifically investigated, research has shown that basal hypersensitivity in response to mechanical stimulation of muscles or viscera can occur in adult offspring following repeated postnatal stress, via maternal separation or exposure to limited bedding material [6], [52], [53]. The impact of SSRIs during development on basal sensory thresholds has not been widely investigated. Our observation of unaltered thresholds to mechanical and thermal stimuli in fluoxetine-exposed offspring is in line with earlier findings describing unaltered thermal thresholds in adult offspring (at P70) following oral fluoxetine administration to non-stressed pregnant mothers [54]. However, this is in contrast with previous work showing that administering fluoxetine directly to neonatal pups via daily injections increased thermal threshold in adolescent rats (at P35) [55]. Thus, basal nociception outcomes may be dependent on the perinatal stress model, route of SSRI administration or behavioral test used.

## Conclusions

The present study is the first to investigate long-term effects of combined prenatal maternal stress and developmental fluoxetine exposure. We show here that both prenatal stress and developmental fluoxetine exposure affect the intensity of post-operative pain differentially in offspring at the age of 8 weeks. Maternal stress decreases and developmental fluoxetine increases post-operative pain hypersensitivity at this age. However, when maternally stressed dams were treated with fluoxetine offspring showed normal post-operative pain profiles, thus SSRI treatment for maternal adversity may normalize nociception in adult offspring. These changes were paralleled, in part, by modulation of the HPA system. Clearly there is a marked long-term effect of maternal adversity and developmental SSRI exposure on post-operative pain in offspring. However, before conclusions can be made about the benefits and risks of perinatal SSRI exposure on offspring development further research is needed.
